# Prevalence and associated factors of binge eating disorder among Bahraini youth and young adults: a cross-sectional study in a self-selected convenience sample

**DOI:** 10.1186/s40337-022-00726-3

**Published:** 2023-01-10

**Authors:** Zahraa A. Rasool Abbas Abdulla, Hend Omar Almahmood, Razan Raed Alghasra, Zahra Abdulameer Sadeq Alherz, Husain A. Ghaffar Alsharifa, Seham Jamal Qamber, Nadia Aaref Alomar, Fatema Ebrahim Almajed, Taher Reyadh Almahroos, Zainab Ali Alnajjas, Adel Salman Alsayyad

**Affiliations:** 1 College of Medicine and Medical Sciences, Arabian Gulf University, Manama, 329 Bahrain; 2 Family and Community Medicine Department, College of Medicine and Medical Sciences, Arabian Gulf University, Manama, 329 Bahrain

**Keywords:** Binge eating disorder, Risk factors, Obesity, Depression, Anxiety, Young adults, Youth

## Abstract

**Background:**

Binge eating disorder (BED) is defined as recurrent ingestion of an unusually large amount of food in a discrete period of time. BED has the highest prevalence of all eating disorders. Studies have shown a strong relationship between BED and both physical and psychological factors such as obesity, depression and anxiety. This research aimed to report the prevalence and associated factors of BED among Bahrainis (aged 15–30 years).

**Methods:**

A total of 959 participants (aged 15–30 years) completed self-administered online questionnaires. BED was measured using the binge eating disorder Screener-7. The Patient Health Questionnaire-9 and General Anxiety Disorder-7 were used to measure depression and anxiety, respectively.

**Results:**

Out of all participants, 21.2% had binge eating symptoms. A higher BMI, a restricted diet, depression and anxiety were associated with more frequent binge eating symptoms. Out of all associated factors, depression had the strongest association with binge eating, (*r*_p_ = 0.371, *p* < 0.0001). However, sociodemographic variables including age and other medical conditions were not significantly associated with BED symptoms.

**Conclusion:**

In conclusion, the prevalence of BED symptoms was significantly high among the study participants. The results point out the crucial role of awareness of the interaction between obesity, depression and anxiety as potential risk factors for binge eating tendencies. Further research should examine their relationship with BED.

## Plain English summary

Binge eating disorder is an eating disorder characterized by the consumption of a large amount of food in a short period of time with loss of control over stopping accompanied by emotional stress during the episode. Studies have identified multiple risk factors that may contribute to binge eating, including lifestyle, psychological well-being of the individual, stress, genetics, family history, age and sex. The current study examined binge eating symptoms among adolescents and young adults aged 15–30 years to estimate the prevalence of the disorder in the Kingdom of Bahrain. Furthermore, participants were tested for depression and anxiety to investigate whether they were associated with the disorder. The results revealed that approximately one-fifth of the participants had binge eating symptoms. A high body mass index, depression and anxiety were significantly associated with binge eating symptoms. We hope this study will be reliable for use in epidemiological studies and further research.

## Background

Eating disorders (EDs) are psychiatric disturbances characterized by abnormal or disturbed eating behaviors that are associated with medical and psychological complications. The Diagnostic and Statistical Manual of Mental Disorders (DSM-5) currently classifies eating disorders into three primary disorders: anorexia nervosa (AN), bulimia nervosa (BN) and binge eating disorder (BED) [1].

BED is defined as recurrent ingestion of an unusually large amount of food in a discrete period of time (e.g., a 2 h period). The DSM-5 diagnostic criteria for BED include a lack of control over the urge to overeat, a minimum of one episode of binge eating per week for three consecutive months, and feelings of marked distress and guilt. Furthermore, three or more characteristics are associated with BED: (i) eating rapidly, (ii) eating when not hungry, (iii) eating until uncomfortably full, (iv) eating in secrecy; to avoid being embarrassed, and (v) negative emotions after overeating. The severity of the disorder, which is determined by the number of episodes per week, has 4 classes: mild, moderate, severe and extreme. Unlike AN and BN, BED individuals are not concerned with their body image as a core or mandatory diagnostic criterion, but it may be present and not followed by compensatory actions [1–3].

BED peaks in later adolescence and young adulthood [4]. According to some prospective studies, uncontrolled eating with bingeing nature during childhood, whether objective or subjective, predicts later development of BED and may lead to excess weight gain and metabolic dysfunction in susceptible youth [5].

BED has the highest prevalence of all EDs [6]. A study was conducted on Saudi women to measure abnormal binge eating attitudes reported a prevalence of BED of 18.1% [7]. Additionally, in Palestine, a study aimed at measuring the prevalence of BED among female college students showed that 50% of the participants had binge eating symptoms [8].

BED is known to be more prevalent in women than in men [9]. Low self-esteem linked with body image is a well-established risk factor predicting the potentiality of BED in women than in men [10]. In particular, the likelihood of women having body image dissatisfaction, low self-esteem and perfectionism is higher than that of males. Constant worries and excessive concerns about error occurrence may induce binge eating behavior [11].

Since the discovery of oil, a substantial sociocultural revolution has taken place in the Arabian Gulf countries, resulting in new dietary habits which in turn are believed to be the reason behind the gradual increase in noncommunicable diseases, including EDs. Moreover, Western cultural exposure, especially through social media platforms, sets specific criteria for an ideal body image that displays a greater risk for psychiatric disorders, such as EDs [12]. It is believed that cultural factors attribute to the increased risk of EDs, e.g., peer and familial pressure regarding an individual’s physical appearance [8].

The association between BED and obesity has become more pronounced: the prevalence of BED was found to increase with the degree of obesity [13]. BED is repeatedly linked to considerable weight gain, and it is thought to result from both uncontrolled compulsive eating and binge eating without compensatory behaviors [14], predisposing individuals with BED to obesity and its physical and psychological health problems. In two studies, Latinos with a Body Mass Index (BMI) greater than 40 and Asian Americans with a BMI greater than 30 were found to have a significantly higher lifetime risk of BED [15].

Studies focusing on obesity and BED were carried out in the Kingdom of Saudi Arabia. One study included women receiving treatments for obesity, and 19%-69% had binge eating episodes. Another study was carried out with a sample classified according to each candidate’s BMI and obesity stage; of all the participants with binge eating symptoms, 23.5% were severely obese and 19.2% were mild to moderately obese [7]. Consequently, obese participants showed increased appetite and abnormal eating attitudes under emotional conditions and stress that were in relation to their symptoms of BED.

BED appears to be preceded by multiple mental, physical, and social risk factors that are widely shared with other EDs [4]. Formal genetic studies indicated that familial and genetic factors were risk factors for BED [15]. A review of the BED literature in the clinical setting showed that approximately 30–80% of individuals who binge eat have lifetime comorbid anxiety or mood disorders. Furthermore, BED significantly raises the risk of developing major depression, bipolar disorder, substance abuse and obsessive–compulsive disorder [16]. Compared to people who are not experiencing EDs, individuals with BED specifically are at higher risk of developing some psychological impairments that could be related to autonomy, environmental mastery, self-esteem and emotional regulation [8]. According to a systematic review of the literature, a significant relationship between BED and depression was found in 10 of 14 studies [17]. Depression is considered both a risk factor for BED and a potential cause [4]. The association between the severity of depressive symptoms and the likelihood of responding to group cognitive behavioral therapy among people with BED has been studied, and the results showed that participants with mild or no depressive symptoms were more likely to respond to therapy than those with severe depressive symptoms [18]. Another study conducted in South Korea on the relationship between BED and depressive symptoms showed that nurses with BED were 1.8 times more likely to have more severe depressive symptoms [19]. This emphasizes that depressive symptoms are considered risk factors for BED and vice versa. To further prove this, a study designed to evaluate the bidirectional association between depressive symptoms and eating disorders in early adolescence was carried out and showed that there was a reciprocal association between binge eating and depressive symptoms [20]. Moreover, another study of women enrolled in weight loss programs showed that participants with BED tended to suffer from more severe depression than those who did not have BED [21]. The same study also concluded that participants with higher BED scores showed more severe depressive symptoms [21].

Anxiety disorders are found in 30–80% of people who have BED [8]. A study conducted in Brazil showed a direct relationship between anxiety and BED symptoms. The same study showed that individuals with anxiety were more likely to develop severe BED symptoms [22]. Another study aimed to examine the relationship between insomnia symptoms and BED in people with an underlying psychiatric condition and showed that the severity of insomnia in patients with BED was significantly higher than that in patients with no history of eating disorders [23]. A study found that BED and BN had moderate/marked functional impairment in work/school, social and family life. Moreover, individuals with BED and BN had significantly high number of days lost from work/school as well as underproductive days at work or school [24].

Furthermore, a study was carried out in a population of patients with diabetes to measure the prevalence rate of overeating symptoms, subclinical binge eating (SBE) and clinical binge eating (CBE) and their association with quality of life, anxiety, depression, HbA1c levels and BMI. The prevalence rates of overeating symptoms, SBE and CBE were 8.4%, 18% and 7.9% respectively. Youth with CBE symptoms scored had the highest scores for anxiety and depression symptoms [25].

BED is associated with a range of medical complications identified in representative community samples. For example, people with BED are at higher risk of developing hypertension along with diabetes mellitus type 2 (DM-2). Moreover, people with BED are more likely to develop metabolic syndrome and dyslipidemia than people who do not suffer from any ED [16]. In a study that examined lifetime medical correlates of eating disorders (AN, BN, and BED), individuals with BED had higher lifetime prevalence of many medical conditions (e.g., diabetes mellitus, hypertension, high cholesterol, high triglyceride, arthritis, sleep problems, and bowel problems) compared to AN and BN groups [26]. Another study displayed a significant association of BED with asthma, arthritis, spine problems, chronic headache, chronic muscle pain, and gastroesophageal reflux independent of individuals body weight [24].

Multiple treatment interventions have been explored for the treatment of BED with varying degrees of support. Cognitive behavior therapy (CBT) and CBT-guided self-help are the two treatment modalities that have shown the best efficacy in terms of the reduction in the frequency of binge eating. However, other proposed options including interpersonal psychotherapy (IPT), selective serotonin reuptake inhibitors (SSRIs), and lisdexamfetamine, have received modest support [27].

Physicians and healthcare professionals show reluctance to diagnose BED despite it being the most common ED [28]. Underestimation of undiagnosed or untreated BED is noticed more in patients who have other associated medical conditions, such as DM-2, metabolic syndrome, and some mental disorders, such as depression or anxiety [16].

The aim of this study was to estimate the prevalence and associated factors of BED in the Kingdom of Bahrain. Moreover, conducting a study in the Middle East, a region where studies of EDs in general are scarce, would be scientifically valuable. The findings of the present study will address this neglected topic and will draw more attention from the public health and primary healthcare authorities in Bahrain to the importance of eating disorders in general and BED in particular to establish screening programs and raise awareness among the population.

## Methods

The present study protocol is reported according to the STROBE checklist [29].

### Study design and participants

This cross-sectional study recruited 959 Bahraini individuals aged between 15 and 30 years old, as eating disorders are more prevalent during late adolescence and early adulthood [30]. Parental consent was required for those under 18 years old to access the questionnaire. During data collection, individuals taking cortisone, and orexigenic drugs, individuals with hormonal disorders and pregnant women were excluded from this study.

### Setting

The data were collected from October 10th to October 24th, of 2020, via a self-administered online questionnaire. The online method was preferred over other survey formats, as online questionnaires can reach people from different socioeconomic backgrounds. In addition, individuals who are not enrolled in schools or universities, and those who are unemployed or desirably do not work, e.g., housewives, can be still reached through online surveys. Moreover, online surveys are more convenient, and a larger sample size can be obtained in a shorter period of time. The online questionnaire was distributed through multiple social media platforms, including WhatsApp, Instagram, Facebook, and Twitter. Three influencers on these social media platforms were contacted to share the questionnaire to gain more responses as adolescents and young adults, especially the targeted group, made up a substantial proportion of their social media followers based on the type of content they shared. One influencer is an actor and an active content creator, one is a doctor, and one is a local football player; all of them had more than 11,000 followers on Instagram.

### Outcome measures

The questionnaire was created using Google Forms. Participants were allowed to choose their preferred language (English/Arabic) to answer the questionnaire. The first section of the form consisted of a brief introduction, the study aim and objectives, data confidentiality, the questionnaire sections, and a consent question. Only individuals who agreed to participate were directed to the next section. In case of any inquiries regarding this research, the study group’s email was also included.

Data from individuals who completed the questionnaire were analyzed solely.

The second part of the form included questions about the sociodemographic information, age and sex to examine their association with BED; both age and sex are considered risk factors for BED, as mentioned previously in the literature. Nationality was used to exclude non-Bahraini participants, as this study was meant to measure BED prevalence and associated factors in Bahrainis. Additionally, data on marital status, educational level, occupation and monthly income were obtained, as some studies demonstrated an association between these variables and BED, e.g., high-income individuals and individuals with occupational stress have an increased risk of BED, as the former have easier access to food, and the latter were found to use binge eating as a coping mechanism for stress [31]. Smoking and eating habits were part of the sociodemographic information; smoking has been shown to be associated with BED [32], and individuals on restricted diets have a higher risk of developing BED [33]. Participants were asked to self-report their height, weight and medical history—certain diseases are linked to a higher risk of BED [34].

To illustrate the relationship between BED and certain risk factors/complications, the BMI was calculated using participants own anthropometric measurements (weight and height), and they were classified following the standard method according to the World Health Organization (WHO) cutoff point [35].

The third part of the questionnaire screened for Binge Eating symptoms. This was done using the Binge Eating Disorder Screener-7 (BEDS-7). Permission was obtained from the original authors of of the BEDS-7 for its translation and use in this study. BEDS-7 is a self-reported screening tool that is designed to detect symptoms of BED rather than making a diagnosis. It was validated according to the DSM-5 diagnostic criteria.

The BEDS-7 is made up of 7 items that focus on episodes of overeating during the last 3 months. To estimate the extent to which the participants had experienced each item over the past 3 months, they were asked to use a 4-point combined severity/frequency scale to answer each question. The scale options were: 0 (never or rarely), 1 (sometimes), 2 (often), and to 3 (always). The total score for each individual was calculated by summing the scores of the 7 items of the BEDS-7. Individuals were considered to have normal eating habits if their total score was less than 5, while individuals with a total score of 5 or more indicated the presence of binge eating symptoms.

Psychological risk factors such as depression and anxiety were evaluated in the fourth and fifth parts of the questionnaire to study the association between depression and anxiety and the risk of developing BED. Validated Arabic-translated versions of the Patient Health Questionnaire-9 (PHQ-9) and Generalized Anxiety Disorder-7 (GAD-7) were used. The PHQ-9 is a self-report instrument with 9 questions that evaluate depression. The total possible score for the nine items ranges from 0 to 27. Depending on the case, the PHQ-9 depression severity score varies; scores of 5, 10, 15, and 20 represent the cutoff points for mild, moderate, moderately severe, and severe depression, respectively. Anxiety was measured using the GAD-7 self-report instrument, which consists of 7 questions. The total score for the seven items ranges from 0 to 21. Scores of 5, 10, and 15 represent ‘mild’, ‘moderate’, and ‘severe’ anxiety, respectively.

### Study size

The following equation was used to calculate the sample size:

$$n = \frac{{Z^{2}_{{\left( {1 - \frac{\alpha }{2}} \right)}} \times \hat{p}\left( {1 - \hat{p}} \right)}}{{E^{2} }}$$

$$n = \frac{{\left. {\left( {1.96} \right)^{2} \times 0. 1516 \times (1 - 0.1516} \right)}}{{0.025^{2} }} = 790.554812 \approx 791$$

${\upalpha }$ = significance level = 0.05 → $\frac{{{\alpha }}}{2}$ = 0.025, 1 − $\frac{{{\alpha }}}{2}$ = 0.975, Z (0.975) = 1.96; p̂ = sample proportion = 15.16%; $E$ = margin of error = 0.025 probability of rejecting the null hypothesis when the null hypothesis is true.

### Statistical analysis

The collected data were statistically analyzed and organized using the Statistical Package for Social Sciences (SPSS) version 23. PHQ-9 and GAD-7 scores were categorized into two categories: (1) scores < 5 indicated normal to minimal symptoms and (2) scores ≥ 5 indicated mild to severe symptoms. We have used the cutoff points which represent the line between the normal and the abnormal given that it would be statistically more robust to find the difference between the two groups rather than categorizing the outcomes based on the questionnaire’s cutoff points. In both questionnaires (PHQ-9 and GAD-7) this cutoff point was 5. Chronic diseases were categorized into three groups: (1) gastrointestinal diseases, (2) cardiovascular diseases and (3) endocrinological diseases.

Categorial variables are expressed as frequencies and were calculated as descriptive statistics, and numerical variables are expressed as the mean ± standard deviation.

The association between the binge eating score and the categorical variables was determined using the independent sample t-test. Pearson’s correlation (*r*_p_) was also used to analyze the correlation between BED and the following variables: BMI, GAD-7 score and PHQ-9 score. A *p* < 0.05 was the statistical significance threshold for this study.

## Results

This research aimed to study the prevalence of BED and its relationship with selected variables among Bahraini youth and young adults. A total of 959 subjects were included in the study.

The distribution of age across the study is shown in Table 1. Most of the study sample are of those between 19 and 22 years old (42.4%) followed by those between 15 and 18 years old (31.1%). Table 2 summarizes the sociodemographic characteristics of the sample. The mean age of the sample was 20.77 SD 3.63 years. The distribution of the participants, 674 (70.3%) were female, and 830 (86.5%) were single. Most of our participants were students (710; 74%). Regarding the educational level, 564 (58.8%) participants had received their high school diploma and 334 (34.8%) had a college degree. There were no major differences between the levels of monthly income among the individuals involved in the study. Most of our participants were non-smokers (803; 83.7%).

**Table 1:** Age categories of Bahraini youth and young adults (n = 959)

Age categories (years)	n	%
15–18	298	31.1
19–22	407	42.4
23–26	165	17.2
27–30	89	9.3

**Table 2:** Sociodemographic data of Bahraini youth and young adults (n = 959)

	Mean ± SD
Age	20.770 ± 3.6282

	N	%
*BMI categories*		
Underweight (< 18.5)	126	13.1
Normal (18.5–24.9)	511	53.3
Overweight (25–29.9)	196	20.4
Obese (> 30)	126	13.1
*Sex*		
Male	285	29.7
Female	674	70.3
*Marital status*		
Single	830	86.5
Married	123	12.8
Divorced	4	0.4
Widowed	2	0.2
*Occupation*		
Student	710	74
Employee	137	14.3
Unemployed (looking for a job)	98	10.2
I do not work	14	1.5
*Education level*		
Primary	2	0.2
Intermediate	59	6.2
Secondary	564	58.8
College	334	34.8
Monthly income*		
< 500 BHD	378	39.4
500–700 BHD	233	24.3
> 700 BHD	348	36.3
*Smoking*		
Yes	156	16.3
No	803	83.7
*On a diet*		
Yes, in the past	224	23.4
Currently on a diet	182	19
No	553	57.7

*BMI* Body mass index

*NB: 500 BD is equivalent to 1327 US dollars and 700 BD is equivalent 1857 US dollars

Table 3 shows the different variables, their mean BEDS-7 scores and their *p* values. There was no statistical correlation between the BEDS-7 score and the following demographic factors; age, marital status, occupation, level of education, monthly income and smoking status (*p* > 0.05).

**Table 3:** The association between the BEDS-7 score and different variables

Variable	BEDS-7 score, mean ± SD	*p* value
*Age*	–	0.718
*Sex*		
Female	2.03 ± 3.232	0.005
Male	1.42 ± 2.485	
*BMI*		
Underweight	0.51 ± 1.770	< 0.0001
Normal	1.55 ± 2.805	
Overweight	2.4 ± 3.286	
Obese	3.51 ± 3.655	
*On a Diet*		
Yes, in the past	2.63 ± 3.371	< 0.0001
Currently on a diet	1.14 ± 2.41	
No	2.94 ± 3.63	
*Total PHQ-9 score*		
Normal–minimal	0.84 ± 1.965	< 0.0001
Abnormal (mild, moderate, moderately severe, severe	2.27 ± 3.303	
*Total GAD-7 score*		
Normal–minimal	0.89 ± 2.163	< 0.0001
Abnormal (mild, moderate, severe)	2.38 ± 3.318	
*Gastrointestinal diseases*		
Present	2.64 ± 3.982	0.521
Absent	1.84 ± 3.030	
*Cardiovascular diseases*		
Present	2.22 ± 3.490	0.648
Absent	1.84 ± 3.033	
*Endocrine diseases*		
Present	2.96 ± 3.649	0.151
Absent	1.82 ± 3.021	

*BMI* Body mass index*, GAD-7* General Anxiety Disorder-7*, PHQ-9* Patient Health Questionnaire-9

The prevalence of positive binge eating symptoms was 21.2% among all participants, as demonstrated in Table 4. The mean total binge score of the whole sample was 1.84 SD 3.04.

**Table 4:** BED-7, GAD-7 and PHQ-9 scores

	n	%
*BEDS-7*		
Normal	756	78.8
Abnormal	203	21.2
*GAD-7*		
Normal–Minimal	344	35.9
Mild	317	33.1
Moderate	174	18.1
Severe	124	12.9
*PHQ-9*		
Normal–Minimal	283	29.5
Mild	325	33.9
Moderate	191	19.9
Moderately severe	103	10.7
Severe	57	5.9

*BEDS-7* Binge Eating Disorder Screener-7*, GAD-7* General Anxiety Disorder-7*, PHQ-9* Patient Health Questionnaire-9

The mean BEDS-7 score of the female participants (2.03 SD 3.23) was higher than that of the male participants (1.42 SD 2.49, with a statistically significant correlation with the BEDS-7 score (*p* = 0.005).

The mean BMI of our sample was 23.88 ± 5.7 kg/m^2^, with approximately 53.3% of the participants in the normal BMI category (18.5–24.9 kg/m^2^). Furthermore, an increase in the BMI category was found to be significantly correlated with a higher mean BEDS-7 score; the mean BEDS-7 score for the obese category was 3.51 ± 3.66, while the mean BEDS-7 score was lower in all other BMI categories.

More than half of the sample (57.7%) was not on dietary restrictions. Significantly higher mean of BEDS-7 scores were found in participants who were previously/currently on a restricted diet compared to those who were not. Dietary restrictions were positively correlated with the BEDS-7 score.

To determine the risk factors for BED, we calculated the scores of depression and anxiety in the sample. Of the whole sample, 283 (29.5%) participants scored below 5 which is considered normal according to the PHQ-9 scale while 676 (70.5%) participants had abnormal PHQ-9 scores ranging from mild, moderate, moderately severe and severe depressive symptoms. According to the GAD-7 score, 344 (35.9%) participants were found to have normal scores, and 615 (64.1%) had abnormal scores ranging from mild, moderate and severe anxiety symptoms (Table 4).

Significantly higher mean BEDS-7 scores were found among participants who had depressive and anxiety symptoms. On the other hand, there was no statistical correlation between the participant’s BEDS-7 score and gastrointestinal, cardiovascular or endocrine diseases.

We studied the correlation between the BEDS-7 score and BED risk factors: obesity, anxiety and depression. The results showed a quantitative correlation between the BEDS-7 score and all the previously mentioned factors. In general, depression had the highest correlation with the BEDS-7 score (*r*_p_ = 0.371, *p* < 0.0001) (Table 5).

**Table 5:** Pearson’s correlation coefficient for BMI, the total anxiety score and the total depression score

Correlations	r	*p* value
BMI	0.287	< 0.0001
GAD-7	0.303	< 0.0001
PHQ-9	0.371	< 0.0001

*BMI Body mass index, GAD-7* General anxiety disorder, *PHQ-9* Patient Health Questionnaire-9

## Discussion

In this study, the prevalence of BED among the participants was 21.2% (n = 203). This finding is in line with other studies; a study in Korea reported that the prevalence was 28%, which was slightly higher than that in our study [30]. A study measuring the predictors of BED in males and females in the UAE found that approximately 24–36% of their study sample reported binge eating [7].

Surprisingly, our study did not find a significant correlation between age and BED, which could be due to different study aims as this study intended to examine the presence of BED symptoms rather than the diagnosis of the disorder. Epidemiological studies have shown that BED is more common in females than males [9], which is similar to the results of our study.

There is a strong association between BED and BMI, and our study found that a higher BMI was associated with a higher BEDS-7 score. Individuals whose BMI was above 30 kg/m^2^ had the highest mean BEDS-7 score. These results were similar to previous studies that found that BED was higher among obese individuals and those who were seeking obesity treatment [36].

Furthermore, in our study, dietary restrictions were positively correlated with the BEDS-7 score, consistent with other studies [37]. This could be explained through different models. It has been hypothesized that the dietary rules followed in a restricted diet become increasingly difficult to maintain over time, and their breakable nature stimulates binge eating. Another hypothesis suggests that hunger and physiological cues intensify food cravings and increase appetite. Another potential theory proposes that the constant attempts to sustain cognitive control over food consumption seem to fail when disinhibitors (e.g., mood fluctuations) disrupt this cognitive control. In summary, dietary restriction is a possible central risk factor and/or maintaining factor for BED [33].

The results of our study showed that high BEDS-7 scores were associated with high scores of depression and anxiety. The multivariable analysis indicated that depression showed a slightly higher correlation than anxiety, and this finding is in line with previous studies. Grilo et al. [38] stated that major depression is the most common comorbidity with BED among other specific mood disorders including anxiety. In general, few data are available on the pathophysiological role of depression in BED. However, a recent hypothesis suggests that the dopaminergic pathway plays a major role in impulsive/compulsive food consumption theory and its regulation by the brain reward system (mesocorticolimbic circuit). Another neuropathway that involves the neurotransmitter serotonin (5-hydroxytryotamine [5-HT]) is thought to play a crucial role in the development of Eds [39]. The first two neurotransmitters, dopamine and serotonin, are a part of the monoamine hypothesis in the pathophysiology of depression [40]. Since BED was classified in the DSM-5 as an ED, different pharmacological treatments have been investigated including two antidepressant drugs (bupropion and vortioxetine) that showed the efficacy of BED treatment during their drug trials. In a small retrospective cohort study, bupropion showed a mood stabilizing effect alongside reductions in binge frequency in patients with mood psychopathological disorders compared to patients with BED who did not suffer from any psychological disorder. Further clinical trials are needed to prove the evident efficacy of antidepressants in improving BED symptoms [39].

Regarding anxiety, its association with BED has been the focus of many recent studies aiming to investigate anxiety and its temporal relationship with BED. People with anxiety experience binge episodes and unconsciously use them as a way to cope with their anxiety, which could be due to their increased appetite when they are anxious. In fact, it has been proven that this coping mechanism is linked to a reduction in anxiety [41, 42]. It has been reported that anxiety disorders are the second most common psychological comorbidity among patients with BED [40]. The literature shows a complex association between anxiety and BED and retrospective studies have shown that anxiety can be a secondary consequence of BED; on the other hand, anxiety can be a risk factor for BED, preceding it with an early onset during childhood [43]. Taking into account the latter association, studies have reported the importance of primary prevention programs as well as early detection of mental illnesses in adolescents to prevent the onset of EDs, particularly BED [44].

## Limitations

To the best of our knowledge, our study is the first to focus solely on BED in the Kingdom of Bahrain. Nevertheless, certain limitations need to be acknowledged for the interpretation of the results. First, the study was a cross-sectional study and it could not determine the relationship between the cause and the effect. Second, we used an online-based questionnaire as our data collection instrument. This method allowed us to obtain a broad sample, yet individuals with limited access to the internet were underrepresented. However, the sample was conveniently selected which may not reflect the characteristics of the general population. Furthermore, since we collected our research data online, we tried to avoid sampling bias by making the survey as short and accessible as possible. The selected influencers created different genres of content to minimize undercoverage bias.

Third, the online survey was not a definitive diagnostic method for BED, which, ideally, is a structured clinical interview. Furthermore, data were collected during the COVID-19 pandemic, which affects the psychological well-being of individuals, whether they have been infected or not. The traumatic impact of the pandemic has resulted in higher percentages of depression and anxiety as well as other psychological disorders [45]. A study carried out in Bahrain during the COVID-19 pandemic showed a high psychological impact on the population [46]. Additionally, although we maximized our efforts to choose influencers that cover different types of content, there was still a potential bias in participant selection. Finally, the majority of the respondents were women. It is possible that a more sex-balanced sample could have enriched the results of the study.

## Conclusions

The present study revealed that the prevalence of BED symptoms among Bahraini youth and young adults was high (21.2%) and it brought attention to the strong association of BED with depression and anxiety.

This signifies the need to develop educational programs to raise the level of awareness of BED and the recognition of its symptoms, along with preventive and screening measures for depression and anxiety. These recommendations must be taken into account and implemented into generated interventional programs. Moreover, primary healthcare professionals, nutritionists and psychologists should come together to improve individuals’ eating habits and diagnose EDs in general and BED in particular. Future repetition of the study is essential to reduce the limitations faced.

## Data Availability

The datasets used and/or analyzed during the current study are available from the corresponding author on reasonable request.

## Abbreviations

BED: Binge eating disorder
ED: Eating disorder
DSM-5: Diagnostic Statistical Manual of Mental disorders, Fifth edition
AN: Anorexia nervosa
BN: Bulimia nervosa
BMI: Body mass index
DM-2: Diabetes mellitus type 2
CBT: Cognitive behavioral therapy
WHO: World Health Organization
BEDS-7: Binge Eating Disorder Screener-7
PHQ-9: Patient Health Questionnaire-9
GAD-7: Generalized Anxiety Disorder-7
SPSS: Statistical Package for Social Sciences
SD: Standard deviation
SBE: Subclinical binge eating
CBE: Clinical binge eating
5-HT: 5-Hydroxytryotamine
COVID-19: Coronavirus disease 2019
